# Managing e-waste from a closed-loop lifecycle perspective: China’s challenges and fund policy redesign

**DOI:** 10.1007/s11356-022-19227-6

**Published:** 2022-02-19

**Authors:** Tingting Tian, Guangfu Liu, Hussein Yasemi, Yang Liu

**Affiliations:** 1 Business Division, Institute of Textiles and Clothing, The Hong Kong Polytechnic University, Hong Kong, China; 2 School of Economics and Management, Tongji University, Shanghai, 200092 China; 3 Department of Management and Engineering, Linköping University, SE-581 83 Linköping, Sweden

**Keywords:** E-waste management, Fund policy redesign, Extended producer responsibility, Sustainability, China

## Abstract

E-waste is one of the fastest growing streams of solid waste globally, and its effective management has become a focused issue, which requires a deep understanding of the core guiding theory of extended producer responsibility (EPR). Over the past 20 years, China, one of the world’s largest producers of electrical and electronic equipment (EEE), has made great efforts to improve e-waste management along with the massive generation of e-waste. In 2012, China implemented a unique EPR-based e-waste fund policy. However, the fund policy is unsustainable due to the challenges of non-closed resource use, informal recycling, and fund imbalance. Beginning with an overview of these challenges, this paper focuses on redesigning the fund policy from a closed-loop lifecycle perspective in order to maintain a balanced development of the resource use loop and the fund system in China’s ten-year plan. In doing so, two EPR instruments, recycling content standards and consumer-oriented deposits, are added to the current fund policy. Subsequently, three extension scenarios alternately changed a critical parameter of the model to test the impact on sustainable capabilities. In this way, the sustainable supply of funds and secondary resources for the e-waste industry can be established in China and effectively demonstrate solid waste management in developing countries.

## Introduction

Over the last few decades, the consumption of electrical and electronic equipment (EEE) has greatly increased with the advancement of technology and the improvement of people’s living standards (Shittu et al. 2021). High consumption is always accompanied by a high yield of waste due to service lifetime limitations (Gu et al. 2016). Approximately 54 million tons of e-waste were generated globally in 2019, and it is speculated that this figure will ascend to 74.7 million tons by 2030, with an average annual growth rate of 3–5% (Arya and Kumar 2020; Ilankoon et al. 2018). E-waste is an attractive “urban mineral” because it contains various valuable resources such as gold, silver, copper, and other precious metal elements (Ogunniyi et al. 2009; Yang et al. 2020). It is an environmental contaminant simultaneously because of its embedded heavy metals, flame retardants, and refrigerants that can pose a severe threat to the environment and human health without proper treatment (Sovacool 2019). Thus, the recovery of these secondary resources from e-waste has significant benefits for resource conservation and pollution prevention (Wang et al. 2019). However, most e-waste is usually “backyard recycled” through illegal and backward informal channels, landfilled, incinerated, or directly dumped (Chi et al. 2011). Only a small amount, less than 20%, of global e-waste is recycled by formal take-back systems (Patil and Ramakrishna 2020).

Under the pressure to minimise the environmental impact of e-waste disposal, many legislative documents have been activated under the guidance of extended producer responsibility (EPR). EPR shifts the physical and/or financial responsibility for e-waste from the municipalities to the original producers, thus providing effective financial and/or physical links between producers and recyclers (Kaffine and O’Reilly 2013). To some extent, EPR is better viewed as a framework within a bound of policies based on three basic categories of instruments: take-back requirements, economic instruments, and performance standards (Kaffine and O’Reilly 2013). Policymakers can select from EPRs that can be flexibly adapted to national and regional values, legislative particularities, or economic contexts. EPR is regarded as a core guiding theory to sustain the development of a recycling-oriented circular economy and has been adopted in many EU countries, the USA, Japan, and China (Gu et al. 2017; Xu et al. 2021). The WEEE Directive (Directive 2012/19/EU) and the RoHS Directive (Directive 2011/65/EU) are the most relevant examples, which emphasise take-back requirements that 65% of electronic equipment sold or 85% of e-waste generated must be recycled (Berežni et al. 2021).

**Fig.1:**
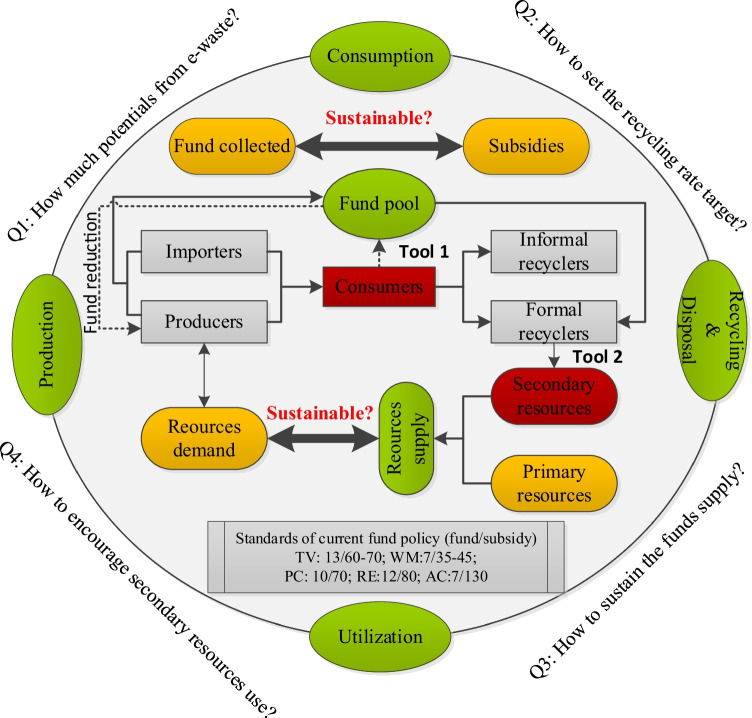
A conceptual framework of redesigned fund policy

China is one of the largest manufacturers and consumers of EEE globally and undoubtedly generates a large amount of e-waste (Liu et al. 2017). According to previous global trends, the USA generated the highest e-waste. However, the latest statistical report shows that China generated more e-waste with 11.17 million tons, followed by the USA with 7.63 million tons, India with 3.56 million tons, Japan with 2.83 million tons, and Brazil with 2.36 million tons in 2019 (Statista 2021). Meanwhile, China’s e-waste generation is predicted to grow to 27.2 million tons by 2030 (Zeng et al. 2020). This situation may be exacerbated if effective measures do not curb it (Li and Achal 2020). To promote e-waste management, in 2012, China issued certificates to 109 formal recyclers and proposed a unique EPR program known as the fund policy (State Taxation Administration of People’s Republic of China 2012). The fund policy assigns financial responsibility to producers for products’ entire life, especially post-consumption, intending to subsidise certified recyclers to increase recycling (Cai et al. 2020). Currently, five categories of electronics, as shown in Fig. 1, including televisions (TV), refrigerators (RE), washing machines (WM), air conditioners (AC), and computers (PC), are on the fund management list with different levies and subsidies standards (Fu et al. 2020). There is no doubt that the fund policy-based e-waste management system plays a key role in the entire life cycle of e-waste in China, but it also faces some challenges that may cause it to stagnate (de Albuquerque et al. 2020).

This paper focuses on the fund policy redesign that provides specific operational strategies for critical links of the entire life cycle based on clarified challenges to manage e-waste dynamically. Accordingly, the contribution of this study to the existing literature is threefold. First, to the best of our knowledge, this is the first study to redesign the e-waste fund policy from a closed-loop lifecycle perspective. Most existing literature investigates the market responses and environmental performance of introducing the fund policy. However, limited literature discussed the availability of sustainable funds and secondary resources throughout the life cycle, which is the focus of this study. Second, our proposed framework provides a new angle for e-waste management by involving two EPR instruments, consumer deposits and recycling content standards, to operationalise closed-loop in both resource and fund flows. Finally, this study creatively compares the feasibility of the redesigned policy under “what if” scenarios of adjusting recycling content standards, recycling rate, and loss rate of precious resources and puts forward corresponding schemes. As such, the redesigned model can flexibly adapt to some changes based on technical levels or regional characteristics and thus is expected to guide policymakers to make better-informed decisions.

The remainder of this study is organised as follows. “A review of China’s e-waste management challenges” provides a literature review of China’s e-waste management challenges under the fund policy. In “The fund policy redesign based on the closed-loop life cycle”, a redesigned framework that integrates two EPR tools (recycling content standards and consumer-oriented deposits) into the current fund policy is proposed. “Results analysis” discusses the case study results to demonstrate the feasibility of the redesign scheme, followed by a sensitivity analysis to test the impact of some critical variables (e.g. recycling resources content, recycling rates, and resource extraction rate). We present management insights and overall concluding remarks in “Discussion” and “Conclusions”.

## A review of China’s e-waste management challenges

Originally, recycling funds intended to internalise the externalities of e-waste treatment in the form of producer/consumer payments (Puig-Ventosa 2004). In current practice, many countries have adopted different fund schemes to support e-waste recycling activities, such as Switzerland, Norway, the Netherlands, Sweden, and Australia. In California, funds levied from consumers are used for recycling infrastructure building, while in Switzerland and Norway, these funds are used to subsidise the recycling costs for recyclers (Nixon and Saphores 2007; Streicher-Porte 2006). Somewhat different in the way it is funded, China’s fund policy collects funds from producers rather than consumers and then subsidises recyclers according to the amount of e-waste recycled (Hischier et al. 2005; Uwasu et al. 2013). Due to the uniqueness of the fund policy, research interest in China’s e-waste management has increased significantly and developed in the past decade (Li et al. 2015; Liu et al. 2021; Zeng et al. 2017). Existing literature discusses the issue of introducing the fund policy-centred management system and investigating the market responses to it (Cao et al. 2016; Yang et al. 2020). In detail, the debate often focuses on how the regulatory regime influences market behaviour or environmental goals, as well as the technical development and economic performance of the e-waste industry (Guo et al. 2018; Liu et al. 2020; Tang and Wang 2014; Wang et al. 2018; Zhang et al. 2015). Arguably, a common view is pointed out in these studies that the current fund policy has not yet met expectations in terms of environmental and economic performance and even faces a series of challenges.

Herein, three main challenges are elucidated from the related publications.

1. Difficulty in closing the resource loop. The fund policy encourages producers to use secondary resources, facilitating source reduction and waste utilisation (Mayyas et al. 2012). Special emphasis is placed on producers paying less if they design new products considering their environmental and natural resource impacts (Gu et al. 2017). However, there are no relevant detailed regulations to enforce it, so it is difficult to incentivise producers to use secondary inputs from e-waste.
2. Competition with informal channels. Informal initiatives exist in most developing countries, especially in China. It is very profitable due to the high demand for secondary resources and the low wages of unskilled workers (Yang et al. 2008). In 2019, only 20% of e-waste was recycled by formal recyclers, whereas the majority was recycled by informal recyclers (Tang and Wang 2014). In Cao et al. (2016)’s study, this proportion has reached 50% in China’s Zhejiang province. The positive contribution of the informal system to waste recycling is acknowledged. However, the environmental problems it causes cannot be ignored, and this issue has been well documented in academia (Botello-Álvarez et al. 2018). It responds only to market demand for high-value waste while leaving others to landfill (Ezeah et al. 2013). This poses a challenge to the stability of e-waste management. Incentives for consumers to give back e-waste to designated collection points are necessary for the long run yet are hard to realise now (Xue et al. 2019). The main reason for this phenomenon is that informal recyclers prefer to pay higher prices than formal recyclers. Take computer waste as an example. In addition to being provided door-to-door service, consumers get 100–150 RMB more if they choose informal channels, which is a great attraction to consumers (Gu et al. 2017).
3. The dilemma of funds supply. The fact that the funds levied from producers are much lower than the subsidies to recyclers leads to a serious dilemma for the fund system: it cannot pay for itself (Gu et al. 2017; Zeng et al. 2017). This paper estimates that the gap between the funds and subsidies was RMB 0.6 billion in 2013 covered by the government. This deficit is likely to increase further as e-waste generation grows and is estimated to grow to RMB 23.15 billion by 2030 (assuming a recycling rate of 0.55). This imbalance betweenfunds and subsidies may lead to a huge burden on the government and impede the sustainable development of e-waste management in China (Liu et al. 2020).

How to address such challenges and further promote the operation of the fund policy-based e-waste management system is an urgent issue. In the study by Gu et al. (2017), a new fund operation mode was established with the addition of consumer payments, and the authors also extended the new model to address the incentive problem of eco-design. Similarly, Hong et al. (2014) described a conceptual fund balance model to determine the socially optimal standards of the funds and subsidies in a closed-loop supply chain. Nevertheless, we should note that the optimal decisions in these studies focus only on piecemeal links and ignore the impacts of other parts throughout the life cycle, which may lead to fragmented or even conflicting decisions. A rational design of environmental regulation policy can promote the extension of producer responsibility (Chen and Sheu 2009). Wang et al. (2018) built an evaluation framework to analyse the effects of China’s fund policy and stressed the need to design a flexible operation mode associated with environmental protection requirements. In this study, we attempt to provide a redesigned scheme of the fund policy that answers the call for a more holistic view. It is hoped that our findings will contribute to assisting policymakers in their task of promoting e-waste management and achieving systematic sustainability.

## The fund policy redesign based on the closed-loop life cycle

This section provides a conceptual framework of redesigned fund policy to demonstrate its operational mechanism clearly and then present the basic data and related models. Due to the lag in statistical data, only historical time-series data of EEE until 2018 can be collected at the time of submission. We use real data between 2007 and 2018 to make future predictions.

### Framework description

In general, the lifecycle phase of EEE flow starts from production by primary producers, flows into social consumption, and then becomes e-waste when it reaches its end-of-life after a certain period (Cucchiella et al. 2015). E-waste is either collected and dismantled by formal/informal recyclers or simply dropped into other solid wastes. In the past, policymakers focused only on the disposal phase rather than the closed-loop life cycle, which led to several serious challenges mentioned before. Since the resource system and the fund system mutually interact, the fund policy should not be introduced in an isolated manner but need to combine incentives for upstream production and downstream recycling from a closed-loop lifecycle perspective. This section attempts to redesign the fund policy from the closed-loop lifecycle perspective, building on the current fund policy with two basic EPR instruments, consumer-oriented deposits, and recycling content standards to achieve long-term dynamic sustainability goals (Kaffine and O’Reilly 2013). Consumer-oriented deposits, also regarded as refunds, are charged at the sales points for EEE and are returned after e-waste is turned into proper collection points (Tojo et al. 2001). Recycling content standards are mandates using specific percentages of secondary resources from e-waste to produce new products (Tojo et al. 2001).

In terms of how this redesign addresses challenges and facilitates the e-waste management system, we can see that (1) connecting the upstream to the downstream of the entire life cycle must rest on the direct effects of resource flows, and recycling content standards alter parts of the virgin resources in favour of closing the resource loop and increasing the amount of e-waste recycled. (2) Consumer deposits increase the fund input channels while placing transactions between consumers and formal recyclers, fundamentally energising the e-waste market. Turning to system goals, it is vital to close the resource use loop by encouraging the use of secondary resources and ensuring a sustainable supply of resources. Apart from this, it is paramount to ensure that adequate funds are available to support related activities, such as incentives for green design, and to adjust the magnitude flexibly rather than fixing them. Figure 1 represents a conceptual framework of this operation approach, which helps to visually illustrate the simple conceptual relationships and how goals and EPR tools can be fit into the product life cycle.

### Models

We propose a reasonable methodology to achieve sustainable development by understanding potential future trends in resources and revenues. The advantage of our models is that they fully consider the changes in product flows over the next decade, ensure that funds can support activities such as subsidies, funding reductions, and deposit returns, and realise the task of optimising the e-waste management system. The operation pathway is divided into four main steps, each of which requires appropriate mathematical models, as follows:

1. Quantitative evaluation of each selected e-waste generation (“EEE obsolete quantity estimation model”).
2. Sustainable resource availability analysis for valuable materials (“Resource supply model”).
3. Sustainable financial availability analysis to support related activities (“Fund supply model”).
4. Sensitivity analysis of key variables to test the feasibility of redesigned models (“Results analysis”).

#### EEE obsolete quantity estimation model

##### Data description

We collected all available data needed for this study. Time-series data on domestic production and imports/exports of EEE were collected for the period 2007–2018 from the China Statistical Yearbook (China NBS 2007–2018). Data on the composition and market prices of specific resources contained in e-waste were obtained from related literature and websites (Akcil et al. 2015, Li et al. 2008, Parajuly and Wenzel 2017, U.S. Geological Survey 2010–2019, Zeng et al. 2016). Accordingly, the fund policy model is redesigned, specifically including an EEE sales model, an obsolete quantity estimation model, a resource supply model, and a fund supply model. For a clearer introduction, the parameters involved in the models are explained in detail, as shown in Table 1.

**Table 1:** Explanation of parameters

${Q}_{ij}$	Consumption quantity of EEE	${\rho }_{ij}$	Green product ratio
${P}_{ij}$	Domestic production quantity of EEE	${r}_{i}$	Recycling rate
${E}_{ij}$	Exports quantity of EEE	${l}_{ki}$	Extraction loss rate
${I}_{ij}$	Imports quantity of EEE	${\theta }_{ki}$	The ${k}_{th}$ resource amount embedded in a product
${G}_{ij}$	Generation quantity of e-waste	${F}_{ij}$	Sustainability of fund supply
$f\left(\mathrm{t}; \beta , \vartheta \right)$	Weibull density function	${f}_{i}$	Fund standard
${\gamma }_{i}$	Refund participation rate	${c}_{i}$	Consumer deposit
${R}_{ij}$	Sustainability of secondary resource supply	${s}_{i}$	Subsidy standard
${\tau }_{ki}$	Recycling content standard	$i,j,k$	$i$, product category; $j$, year; $k,$ resource type

##### EEE sales model

It is essential to know the quantity of EEE consumption ${Q}_{ij}$, which is attributed to domestic production ${P}_{ij}$, exports ${E}_{ij}$, and imports ${I}_{ij}$. Therefore, the total consumption amount of EEE of category $i$ in year $j$ can be defined by Eq. 1.

1 $${Q}_{ij}={P}_{ij}+{I}_{ij}-{E}_{ij}$$

##### Obsolete quantity estimation model

As mentioned before, EEE becomes fatigued after being used repeatedly during their lifetime and then flows out as e-waste. This phenomenon can be described by the Weibull distribution, which has been adopted for e-waste estimation in many precious studies and is considered the most available method (Balde et al. 2015). Accordingly, the Weibull statistical distribution is used in this study to model the estimation of e-waste (Mueller et al. 2007). In particular, the probability density function (Weibull and Rockey 1962) is given by Eq. 2.

2 $$f\left(t; \beta , \vartheta \right)=\left\{\begin{array}{c}\frac{\beta }{\vartheta }{\left(\frac{t}{\vartheta }\right)}^{\beta -1}{e}^{{-\left(x/\vartheta \right)}^{\beta }} t\ge 0\\ 0 t<0\end{array}\right.$$

where $\beta$ is the shape parameter $(\beta >0)$, which determines the shape of the Weibull density function, and $\vartheta$ is the scale parameter $(\vartheta >0)$, which represents the corresponding position of the peak of the function graph on the horizontal axis.

Based on the Weibull density function $f\left(t; \beta , \vartheta \right)$ and the EEE consumption ${Q}_{ij}$, we set $t=j-2007$, then the e-waste ${G}_{ij}$ generated in year $j$ can be determined by Eq. 3.

3 $${G}_{ij}={f}_{i}\left(t\right){Q}_{i,j-t}={f}_{i}\left(j-2007\right)\times {Q}_{i,2007}+{f}_{i}\left(j-2008\right)\times {Q}_{i,2008}+\cdots +{f}_{i}\left(1\right)\times {Q}_{i,j-1}$$

#### Resource supply model

Resources contained in e-waste can obviously provide a choice to supply resources of the EEE industry, as many studies proposed. According to the model from the literature (Sugiyama and Koonsed 2017), in order to ensure a sustainable resource supply for the EEE industry in the future 10 years, based on the recycling content standard, we define the sustainability of the secondary resource supply as ${R}_{ij}$ to establish a sustainable resource supply model (from 2021 to 2030), as in Eq. 4.

4 $${R}_{ij}=\left({(1-l}_{ki}){{r}_{i}G}_{ij}-{\rho }_{ij}{Q}_{ij}{\tau }_{ki}\right){\theta }_{ki}$$

In this model, we focus on the measurement of two indicators. First, we estimate the demand for secondary resources in the EEE industry and set the recycling content standard which is ${\tau }_{ki}$. Since the EEE industry can use natural resources in addition to secondary resources, green and nongreen products coexist in the EEE market. Unlike nongreen products that use all primary resources, green products contain secondary resources that meet the recycling content standard and reach the quality of primary resources. We assume that the proportion of green products is ${\rho }_{ij}$ and the amount of ${k}_{th}$ resource embedded in a product is ${\theta }_{ki}$. Second, we measure the secondary resource supply capacity of e-waste. Technology and equipment are significantly important for extracting secondary resources, but this is still a vital obstacle in developing countries, especially China. This means that the loss rate of precious resources should be considered, and we assume it is ${l}_{ki}$ in our case.

The resource supply model, together with the recycling content standard ${\tau }_{ki}$ and other parameters, can provide the government with management information, such as the recycling rate ${r}_{i}$. If it is likely to keep a balance of secondary resources, namely, ${R}_{ij}\ge 0$, then we can obtain that the recycling rate ${r}_{i}$ should satisfy a certain condition: ${r}_{i}\in \left[\underset{j}{\mathrm{max}}\left(\frac{{\rho }_{ij}{Q}_{ij}{\tau }_{ki}}{(1-{l}_{ki}{)G}_{ij}}\right),1\right]$.

#### Fund supply model

Arguably, a common theme in some articles presents a view that China’s e-waste fund policy—along with support schemes—has encountered financial obstacles that, as mentioned before, have resulted in less-than-expected environmental and economic performance. In a study by Yu et al. (2010), where China’s e-waste pilot projects are studied, the authors stressed the need to redesign management policies and proposed that deposits be returned to consumers as an incentive to surrender e-waste. Subsequently, Gu et al. (2017) redesigned the e-waste fund mode by adding consumer pay parts as a financing way to achieve a sustainable fund system, which inspires our case. But the difference is that in this study, we advocate and attempt to provide more systematic and flexible thinking by adding a consumer-oriented deposit measure rather than simple consumer payment to achieve better financial sustainability ${F}_{ij}$ with higher take-back rate requirements. The fund supply model can be represented by Eq. 5.

5 $${F}_{ij}={(f}_{i}+{c}_{i}-{\rho }_{ij}{f}_{i}){Q}_{ij}-\left({s}_{i}+{\gamma }_{i}{c}_{i}\right){{r}_{i}G}_{ij}$$

The deposits ${c}_{i}$ is commonly used to encourage refund participation (Mallawarachchi and Karunasena 2012). We set the refund participation rate to ${\gamma }_{i}$. Note that in order to reroute e-waste from the informal to the formal sector, the numerical value of the deposit should be set high enough to encourage participation but not so high as to affect consumers’ ability to purchase EEE adversely. According to Eq. 5, the satisfaction condition of the consumer deposit can be obtained: ${c}_{i}\ge \underset{j}{\mathrm{max}}\left(\frac{{s}_{i}{r}_{i}{G}_{ij}-\left(1-{\rho }_{ij}\right){f}_{i}{Q}_{ij}}{{Q}_{ij}-{\gamma }_{i}{r}_{i}{G}_{ij}}\right)$.

## Results analysis

This section first examines the future trend in e-waste and total funds in–out. Since the current fund policy was implemented in 2012, we show the results for the period 2012–2030. Next, we perform a basic scenario analysis of the redesigned fund policy using TV waste as a case study and then conduct sensitivity analysis with four additional scenarios.

### E-waste tendency under the current fund policy

**Fig. 2:**
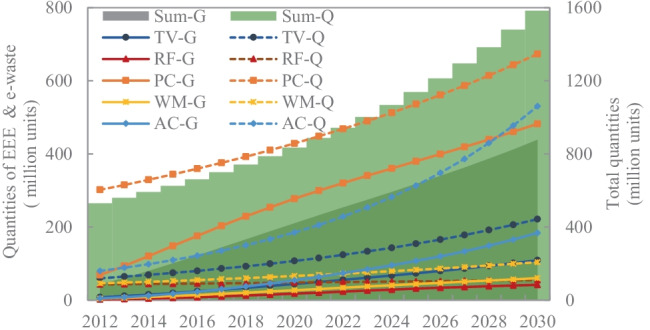
Individual and total quantities of EEE and e-waste

**Fig. 3:**
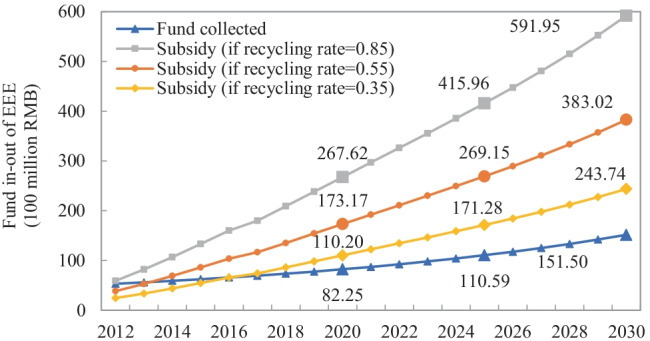
Total fund in–out of EEE under fund policy

The quantities of EEE consumed and e-waste generated in China from 2012 to 2030 are shown in Fig. 2. There is a large gap between the total consumption curve and the total scraped curve, while both grow steadily at an average rate of 10.29% and 11.04%, reaching 876.75 million and 1583.54 million units, respectively, in 2030. These tendencies provide the possibility of realising a sustainable resource supply of the EEE production based on recycled resources from e-waste. In terms of individual streams, the quantities of PC consumption and scraped grow fastest, followed by AC, TV, WM, and RF. The scraped quantity of TV, for instance, rapidly grows from 7.89 million units to 108.83 million units from 2012 to 2030, while the quantity of TV consumed in 2030 is almost four times the 59.85 million units consumed in 2012. Based on the current fund standards, three lines of subsidies are assumed: the upper line ($${r}_{i}=0.85)$$, the baseline ($${r}_{i}=0.55)$$, and the lower line ($${r}_{i}=0.35)$$. As seen in Fig. 3, even with a recycling rate of 0.55, the fund pool will be in deficit by 2023. The higher the recycling rate, the more subsidies and thus the faster the deficit. It indicates that the current fund policy is encountering a financial crisis because of the staggering growth of e-waste, so the redesign of the e-waste management policy should be taken seriously (Zeng et al. 2016).

### Effects of the redesigned fund policy

**Fig. 4:**
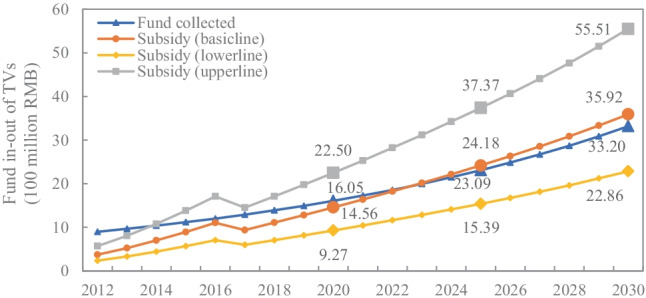
Fund in–out of TV under fund policy

TV waste has always been favoured in the e-waste industry due to its high benefits. In 2019, about 0.84 billion e-waste was processed by 94 certified recyclers in China’s 29 provinces, 51.74% of which were TV (Ministry of Ecology and Environment of People’s Republic of China 2020). According to our prediction, the total amount of funds collected from TV producers cannot cover the subsidies for TV waste at a recycling rate of 0.55 or greater under the current fund policy after 2022, as shown in Fig. 4. Thus, in this section, TV waste is employed as a case study to further analyse the feasibility of the redesigned policy in terms of fund supply and the resource supply. Materials embedded in products can be recycled and repurposed to achieve economic convenience. In fact, the products are usually not homogeneous due to the mix of materials. The profitability of recycling is driven by the inclusion of key and valuable resources, which is a primary consideration for the e-waste industry. Therefore, it is appropriate to make an economic assessment by multiplying the market price by the potential recycling quantity. The purpose is to select the resource category with the highest economic potential for recycling. We can obtain that the most valuable resource in TV is copper, so in this case, the goal is to meet the supply of copper.

**Fig. 5:**
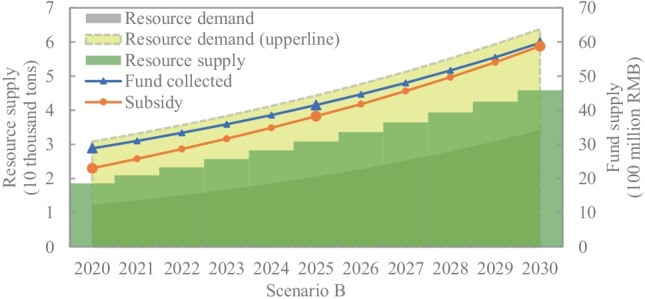
The sustainable capabilities of TV under redesigned policy

The basic parameter values adopted from the existing literature (Chi et al. 2014; Fogarasi et al. 2014; Palmer and Walls 1997; Zeng et al. 2016) are set as follows: ${\theta }_{ki}=824 g/unit, {l}_{ki}=0.07, {\tau }_{ki}=0.35,{\gamma }_{i}=0.70$. In addition, it is thought that with the legal enforcement and ethical standards increase, more producers will practice the use of secondary resources. Hence, it is assumed that the annual growth rate of green products is 3%, with an initial value of 0.40 (Santolaria et al. 2011). According to these basic parameters, we can obtain the standard of the recycling rate ${r}_{i}\in \left[\mathrm{0.42,1}\right]$ and set ${r}_{i}=0.55.$ Correspondingly, the range of the consumer deposit, ${c}_{i}\ge 11.50$, can be obtained, and we set the value of hypothetical group (${r}_{i}, {c}_{i}$) to (0.55, 12) as the basic scenario (scenario B) to illustrate the sustainable capability in our case , as shown in Fig. 5.

The sustainable supply capability of copper in TV is presented in Fig. 5. Due to the consumption growth, the demand for copper in TV shows an upward trend with an increasing rate of 17.79% per year. Meanwhile, the supply of secondary copper from TV waste is increasing, and the surplus is gradually expanding at the assumed recycling rate. This means that the redesigned policy realises a sustainable supply of copper for the TV industry through applying the EPR tool of the recycling content standard. Figure 5 discusses an additional “what if” situation where all TVs, rather than some, contain secondary copper. The result shows that at a 35% recycling content standard, recycled copper cannot satisfy the production needs of all TVs. This emphasises that the recycling rate should cope well with the demand to sustain the resource supply.

In terms of fund supply, with the inclusion of the consumer deposit, we can sustain the fund pool since the funds are higher than subsidies from 2021 to 2030, as shown in Fig. 5. It is noted that in addition to subsidising recyclers, we consider two additional expenditures: (1) rewarding the producers who produce green products with rebate funds and (2) returning deposits to consumers who give back their TV waste. In this way, not only would e-waste be enclosed for resource utilisation, but consumers would also be encouraged to return e-waste to formal collection points due to higher pay, enhancing the competitiveness of formal recyclers. Overall, the redesigned policy could address the challenges of competition posed by informal recyclers and fund deficits, as well as financially support the closure of resource use.

### Sensitivity analysis

**Fig. 6:**
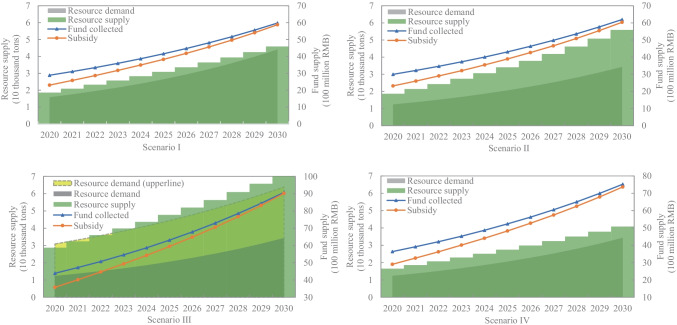
The sustainable capabilities of TV for different scenarios under redesigned policy

In this section, the three key parameters proposed above are chosen as “what if” scenarios to analyse the sensitivity of the redesigned policy compared to scenario B as shown in Fig. 6, which is beneficial for further understanding.

- Scenario I: The recycling content standard is increased to 45%.
- Scenario II: The recycling rate increases from 0.55 at an annual rate of 3%.
- Scenario III: The recycling rate is required to reach 85%, which is the international standard set by the EU (Directive 2012/19/EU).
- Scenario IV: The loss rate of precious resources increases to 30%.

Even though the closed-loop scheme has obtained support from China’s government, the specific plan is still unclear at the moment. In scenario I, we aim to observe whether the redesigned policy can cope with changes in the recycling content standard. When a higher resource content is required, which obviously leads to greater demand, then a new standard of the recycling rate ${r}_{i}\in \left[\mathrm{0.53,1}\right]$ can be obtained. Scenario B still meets this condition. However, it is clear that if a higher recycling content is required, scenario B needs to be further adjusted to maintain the sustainable capacities both of resources and funds.

However, if we directly change the recycling rate with a growth rate of 3%, due to the increase in subsidies, the consumer deposit standard would change from 12 to 13 to maintain the sustainability of the fund system. Scenario I and scenario II illustrate that the recycling rate is positively related to the recycling content standard. Both, in turn, affect the sustainability of the fund system and sometimes require further adjustments to the consumption deposit. Scenario III further confirms that there is a significant increase in the consumer deposit, i.e. ${c}_{i}\ge 25.65$, when the recycling rate increases. Actually, in this case, the supply capability of secondary copper can satisfy the maximum production demand of all TVs rather than only a fraction of them. Thus, we verify the claim that e-waste is urban minerals, and at the same time, we believe that increasing the formal recycling rate is of great significance for achieving resource sustainability.

In scenario IV, reducing the extraction ratio of precious resources has a similar but more significant impact than in scenario I. The difference is that due to the loss of resources, the supply is reduced, while the demand remains constant, requiring a higher recycling rate, i.e. ${r}_{i}\in \left[\mathrm{0.59,1}\right]$. Accordingly, the consumer deposit is enhanced, i.e. ${c}_{i}\ge 18.07$, and we set (${r}_{i}, {c}_{i}$) = (0.70,19) in scenario III to input more resources and funds in order to keep sustainability. Scenario IV does not significantly increase the supply of copper compared to scenario B. Because a large amount of copper is wasted by backward and inefficient treatment means, which often occurs with informal recyclers, this stresses the need to treat e-waste properly in addition to the importance of technological innovation so that e-waste can play an optimal role as an urban mineral for sustainable development.

## Discussion

With our redesigned model, we consider the sustainable resource supply and fund supply of the e-waste fund policy and examine the efficiency of a proper adjustment scheme through four scenarios. It can be found that the current fund policy is not necessarily effective in e-waste management. We argue that the first required adjustment to the fund policy is to include consumer deposits to the fund pool to support higher recycling rates and other economic incentive activities. This scheme would effectively reduce the financial pressure on the government and increase consumers’ motivation to return their e-waste. Only then would the government fully shift financial responsibility to the “producers”. The same government department would best develop the consumer deposit system as the current fund policy, which should also be responsible for the e-waste market system, including cyberinfrastructure maintenance, data management, etc.

Given that recycling content standards are shaped by vigorously promoting circularity, it appears necessary to recognise that policies should mandate a certain percentage of recycled resources for particular consumption goods. For example, the USA enforced newsprint to contain recycled papers. Japan has implemented laws on green purchasing that require the public sector to buy products partly made of recycled resources, such as EEE, office automation machines, and vehicles. Even though China has not yet issued specific implementation schemes, the redesigned fund policy is clearly aimed at improving recycling rates under the recycling content standard, based on “closing the loop” of the product life cycle. However, it may not be good for producers and consumers, as the use of recycled resources can reduce the quality of products relative to virgin material. Thus, producers need to invest more in technological innovations, and consumers should be involved in green consumption.

In doing so, another issue that should be mentioned is consumers’ knowledge and awareness of e-waste. Lack of awareness, insufficient information, moral norms, environmental beliefs, and sociodemographic factors (e.g. age, gender) have been examined significantly affect consumers’ motivation to return e-waste. Thus, the government has a vital role in the efficiency of the redesigned fund policy and has to know how to provide related programs to improve consumers’ attitudes towards e-waste recycling. In fact, consumers demand more information on where they can recycle their e-waste instead of why they should recycle (Islam et al. 2021). Thus, the construction and improvement of proper recycling facilities are the first step in offering available recycling services. Meanwhile, with the skyrocketing online market development, it is necessary to consider e-commerce as an essential element of the e-waste management system. In light of this, the use of the Internet of Things (IoT) and big data analytics could provide new insights into the formal e-waste trade.

In addition, the results of comparative sensitivity analysis can all be adjusted to the characteristics of the different parameters. Obviously, the target recycling rate is positively correlated with the recycling content standard. From the perspective of resource extraction, reducing the loss rate also seems to be a favourable scheme since it avoids resource wastage, which often occurs in informal recycling systems. Therefore, the government can continuously encourage high-tech innovations in resource extraction while carrying out formalisation programs for informal recycling. It is important to note that the standards of the redesigned policy, such as recycling content standards, recycling rates, and consumer deposits, need to be adjusted according to product differences because of the individual treatment of various e-waste types. In the long run, a better scheme would also consider the encouragement of green production, which has been examined in the existing literature (Liu et al. 2020; Zhao and Bai 2021), and appropriate incentives can improve producers’ willingness of green production, and it seems to be another important avenue of e-waste management.

## Conclusions

The e-waste management policy has a substantial impact on the regularisation of the e-waste industry and the utilisation of secondary resources. This study applies EPR theory to redesign the current e-waste fund policy by integrating two typical EPR instruments: recycling content standards and consumer-oriented deposits. In addition to addressing the deficit challenge of the fund system, this approach encourages the use of secondary resources, which is consistent with the original intent of the fund policy. It also highlights the crucial role of consumer behaviour in making formal recyclers more competitive by giving back the e-waste in the form of deposits. Additionally, this study examines the roles of three vital factors—recycling contents standards, recycling rate, and resource extraction rate—in achieving dynamic management.

This study has some limitations. For example, there lack surveys on Chinese consumers’ willingness to deposit, such as the one conducted by Islam et al. (2021), which may be of interest. Although the new model is generic, the situation is different for different e-waste. Due to the limited scale of this study, only TV waste is chosen as a case for policy-made analysis. In addition, future studies could extend the scope to e-waste that is not in the management catalogue, such as small household appliances that have not yet attracted the attention of policymakers.

## Data and materials availability

The datasets used and/or analysed during the current study are available from the corresponding author and first author on reasonable request.
