# Correction to: Managing e‑waste from a closed‑loop lifecycle perspective: China’s challenges and fund policy redesign

**DOI:** 10.1007/s11356-022-19508-0

**Published:** 2022-03-05

**Authors:** Tingting Tian, Guangfu Liu, Hussein Yasemi, Yang Liu

**Affiliations:** 1 Business Division, Institute of Textiles and Clothing, The Hong Kong Polytechnic University, Hong Kong, China; 2 School of Economics and Management, Tongji University, Shanghai, 200092 China; 3 Department of Management and Engineering, Linköping University, SE‑581 83 Linköping, Sweden

**Correction to: Environmental Science and Pollution Research**

**https://doi.org/10.1007/s11356-022-19227-6**

In Equation 2, there should be a space between the formula and constraint.

The Original article has been corrected.

